# Deep-Learning-Based Automated Anomaly Detection of EEGs in Intensive Care Units

**DOI:** 10.3390/bioengineering11050421

**Published:** 2024-04-25

**Authors:** Jacky Chung-Hao Wu, Nien-Chen Liao, Ta-Hsin Yang, Chen-Cheng Hsieh, Jin-An Huang, Yen-Wei Pai, Yi-Jhen Huang, Chieh-Liang Wu, Henry Horng-Shing Lu

**Affiliations:** 1 Institute of Statistics, National Yang Ming Chiao Tung University, Hsinchu 300093, Taiwan; chwu@nycu.edu.tw (J.C.-H.W.); z85021212@gmail.com (T.-H.Y.); astanley414@gmail.com (C.-C.H.); 2 Department of Critical Care Medicine, Taichung Veterans General Hospital, Taichung 407219, Taiwan; rufus0822@gmail.com (N.-C.L.); encourage1009@vghtc.gov.tw (Y.-J.H.); 3 Department of Neurology, Neurological Institute, Taichung Veterans General Hospital, Taichung 407219, Taiwan; jahuang2639@gmail.com (J.-A.H.); jasminezxcv@vghtc.gov.tw (Y.-W.P.); 4 Institute of Clinical Medicine, National Yang Ming Chiao Tung University, Taipei 112304, Taiwan; 5 Department of Health Business Administration, Hungkuang University, Taichung 433304, Taiwan; 6 Department of Post-Baccalaureate Medicine, College of Medicine, National Chung Hsing University, Taichung 402202, Taiwan; 7 Department of Statistics and Data Science, Cornell University, Ithaca, NY 14853, USA

**Keywords:** anomaly detection, EEG, GRU, ICU, intensive care unit, spike

## Abstract

An intensive care unit (ICU) is a special ward in the hospital for patients who require intensive care. It is equipped with many instruments monitoring patients’ vital signs and supported by the medical staff. However, continuous monitoring demands a massive workload of medical care. To ease the burden, we aim to develop an automatic detection model to monitor when brain anomalies occur. In this study, we focus on electroencephalography (EEG), which monitors the brain electroactivity of patients continuously. It is mainly for the diagnosis of brain malfunction. We propose the gated-recurrent-unit-based (GRU-based) model for detecting brain anomalies; it predicts whether the spike or sharp wave happens within a short time window. Based on the banana montage setting, the proposed model exploits characteristics of multiple channels simultaneously to detect anomalies. It is trained, validated, and tested on separated EEG data and achieves more than 90% testing performance on sensitivity, specificity, and balanced accuracy. The proposed anomaly detection model detects the existence of a spike or sharp wave precisely; it will notify the ICU medical staff, who can provide immediate follow-up treatment. Consequently, it can reduce the medical workload in the ICU significantly.

## 1. Introduction

There are many causes of unconscious patients in the intensive care unit (ICU). Figuring out the reason behind it has always been a tricky process. Whether it is blood testing, brain computed tomography, or even MRI, they are all tools that are often used for differential diagnosis. Still, these tools can only represent the current situation, the situation of a point. Continuous electroencephalography (EEG) monitoring is essential for more in-depth tracking of constant changes. Early detection and early treatment are very important milestones in the medical field. We try to use different methods of framing data to achieve the purpose of anomaly detection. According to research statistics for the intensive care unit, 8–37% of patients have had a non-convulsive seizure [1]. Delayed diagnosis or treatment of non-convulsive seizures is associated with a high death rate. Between 10 and 67% of non-convulsive seizures may go undetected without continuous EEG monitoring, and 56% of non-convulsive seizures will be detected within the first hour with continuous EEG monitoring. It has been monitored that 88% of non-convulsive seizures are seen within 24 h. In particular, continuous EEG monitors can detect non-convulsive seizures in patients early, assist physicians in the timely detection of the brain or neurological changes, and provide patients with immediate treatment to prevent permanent damage, which is an essential tool for clinicians in diagnosing disease. Interpreting a large number of EEGs is a very labor-intensive task. The importance of continuous EEG monitoring lies in the early diagnosis of epilepsy and can significantly reduce complications and mortality. About 30% of ICU patients with impaired consciousness have epilepsy, and 90% of them have non-convulsive epilepsy, and only EEGs can make a diagnosis. When the burden of epilepsy is heavier, it means that the damage to the brain will continue to increase over time, which will lead to aggravating epilepsy changes and form a vicious circle. Therefore, immediate treatment becomes very important. In the case of a large amount of continuous EEG data, the clinical side cannot load such a large amount of EEG interpretation. Therefore, the aided performance of artificial intelligence and deep learning is a good choice and development goal.

In this study, we focus on the EEGs characters that are highly related to epilepsy. Using the patients’ EEG data in the past and the doctors’ interpretation and marking can teach the machine to quickly identify abnormal EEGs in subsequent continuous EEG monitoring, thereby improving the efficiency of diagnosis and reducing the burden on the clinical side. The novelty and contributions of this study can be summarized as follows.

- The automated anomaly detection targets patients who are heavily ill and taken care of intensively in the intensive care unit of the Taichung Veterans General Hospital (TCVGH), a national-level medical center, not patients taking some routine and/or physical examinations.
- We attempt to detect anomaly brainwaves before their occurrence so that we can consider possible follow-up treatments in advance. The developed early detection models have promising performance and show great potential in clinical applications.

## 2. Related Works

EEGs have been used to conduct various kinds of research works [2]. The problem of sleep stage classification is studied to help the diagnosis of sleep disorders [3,4] and to measure sleep quality [5]. Some researchers study how to perform automatic emotion recognition [6,7]. The investigations of EEG motor imagery signals have proliferated due to the great potential in brain–computer interface applications [8,9]. The evaluation of mental workload is studied for maintaining working performance and preventing accidents [10,11]. Some researchers have attempted to solve the problem of automatic detection of epileptic seizures, which can be used to improve the patient’s life quality [12], and some have focused on the task of event-related potential detection [13,14].

There has been a large amount of spike detection methods published in the literature [15,16]. The released methods are mainly divided into the following: mimetic analysis [17,18], template matching [19], power spectral analysis [18,20], wavelet analysis [21], and artificial neural networks (ANNs) [22,23,24]. The features obtained from the above methods are seen as input of the methods. In some methods mentioned above, they use their data to fit a classifier. In clinical application, spikes and sharp waves have the same clinical performance when these events happen. As a result, they may be seen as the same class in some papers. Due to the quick development in Graphics Processing Units (GPUs) and Compute Unified Device Architecture (CUDA), a software layer gives direct access to the GPU’s virtual instruction set and parallel computational elements, for the execution of compute kernels [25], and most of the current methods are mainly based on deep learning models [26,27,28,29]. Beyond the task of spike detection for EEGs, the framework of deep learning and transfer learning is now dominating the domain of healthcare in the diagnosis of various diseases and for solving many biomedical problems [30,31]. Applications include, but are not limited to, the automated detection of mycobacterium tuberculosis [32], personalized medicine with electronic health record (EHR) data [33], diagnosis of ophthalmic diseases [34], drug discovery [35], and gene expression classification [36].

Finally, we compare our work with existing work. Due to the differences in the data acquisition and experimental setting, such as the sampling rate and the filter band, it is hard to provide a fair comparison. However, we manage to provide the qualitative comparison in Table 1. In a nutshell, our work targets patients who are heavily ill and taken care of intensively in the ICU of the national-level medical center, TCVGH, not patients taking some routine and/or physical examinations, and it achieves comparable performance with the finest time resolution (i.e., the window size). In addition, we develop early detection models that have promising performance and show great potential in clinical applications, while cross-institutional validation should be conducted in the future to further support and expand the impact.

## 3. Materials and Methods

### 3.1. Working Flow

**Figure 1:**
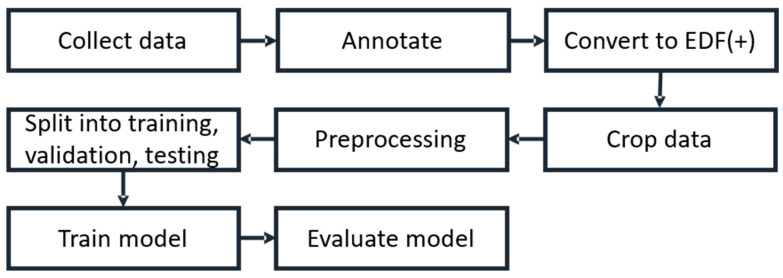
The working process performed in this study.

In this study, the experimental subjects are ICU patients. We focus on patients’ EEG characteristics. Since patients were admitted to ICUs for various reasons, close and intensive care was performed. Many of them suffered from persistent conscious disturbance even though some common causes such as hemodynamic instability and electrolyte imbalance were ruled out. After consulting neurologists, EEG examinations were performed. The working process of this study is shown in Figure 1. Taichung Veterans General Hospital (TCVGH) collected and provided retrospective EEG data recorded from the ICU patients, which are used to train the deep learning models. Medical doctors in TCVGH marked the patients’ EEGs with their or their family’s permission.

### 3.2. ICU Data

**Figure 2:**
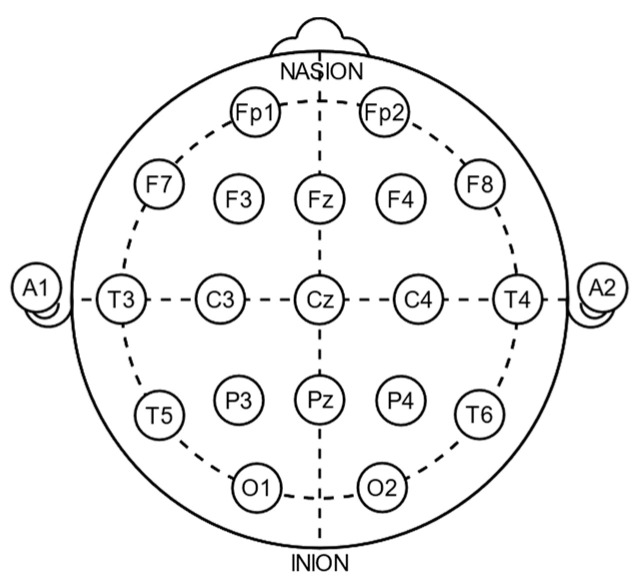
The International 10–20 system for EEG recording. トマトン124, Public domain, via Wikimedia Commons [39].

Electroencephalography (EEG) is a method used to record an electrogram of the discharge of electrodes attached to the scalp. It has become widely accepted for recording activity below the surface of the brain. Because the electrodes are placed along the scalp according to certain methods, such as the International 10–20 system, it is not invasive typically. EEG measures voltage fluctuations resulting from ionic currents within the neurons of the brain. Clinically, EEG refers to the recording of the brain’s spontaneous electrical activity over a period of time, as recorded from multiple electrodes placed on the scalp [37]. Clinical applications usually focus on either event-related potentials or the spectral content of EEG. The former investigates potential fluctuation changes at the moment of the event, such as “eyes open” or “stimulus onset”. The latter analyzes the type of neural oscillations, which is popularly referred to as “brainwaves”, that can be observed in EEG signals in the frequency domain. EEG is widely used in many clinical applications, including sleep disorders, depth of anesthesia, coma, encephalopathies, and brain death. But it is most often used to diagnose epilepsy, which causes abnormalities in EEG readings [38]. Nowadays, EEG mainly uses disc electrodes in clinical practice, and the electrodes are placed according to the International 10–20 system, including 19 recording electrodes and 3 reference electrodes, as shown in Figure 2. Among them, 10 and 20 refer to 10% and 20% of the distance from the nasion to the inion. Fp represents pre-frontal, F represents frontal, C represents central, O represents occipital, and T represents temporal. Also, the mastoid process after ears, A1 and A2, is defined as reference electrodes. The main advantage of using the International 10–20 system is that it can identify the same relative position on the scalp regardless of the size of the head.

EEG data in this study were collected and provided by TCVGH. We use European Data Format (EDF) as the data format to store the EEG recording of ICU patients. EDF is a standard file format designed for the exchange and storage of medical time series [40]. Being an open and non-proprietary format, EDF(+) is commonly used to archive, exchange, and analyze data from commercial devices in a format that is independent of the acquisition system. In this way, the data can be retrieved and analyzed by independent software. EDF(+) software (browsers, checkers, etc.) and example files are freely available [41]. Neurologists put annotations in EDF(+) files. We use the Python language and the MNE package to read EDF(+) and retrieve the information, such as when and where anomaly brainwaves occur. In this study, a total of 8 ICU patients’ records are used to conduct the experiments.

**Figure 3:**
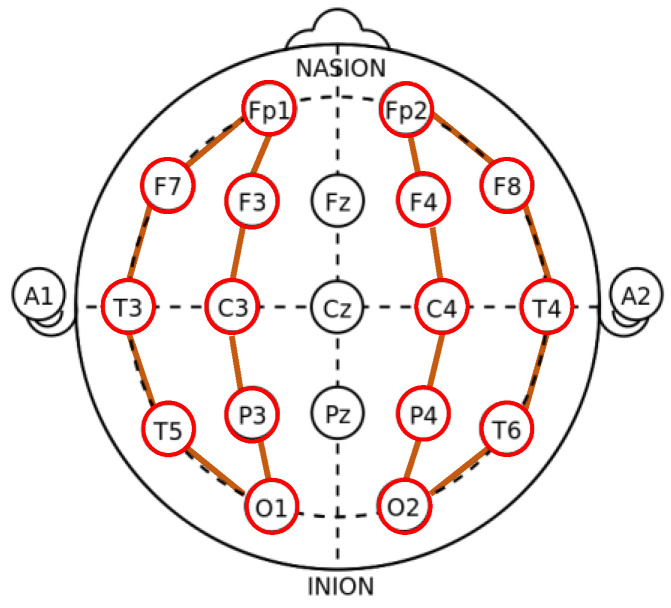
The chains of the bipolar montage. Red circles denote the electrodes and orange lines represent the neighboring relationship between electrodes in clinical practice.

As a general rule, modern montages allow for easy visualization of comparable scalp areas, so they may be assessed for symmetry [42]. There are two primary montages: bipolar montage and monopolar montage. For epilepsy brainwaves, bipolar montage is a better choice to observe its anomaly situation. Medical doctors of TCVGH provided EEG data with bipolar montages which consist of chains of electrodes, each one connected to two neighboring electrodes. The bipolar montage is also called the “banana montage”. Its transverse montage links adjacent electrodes in a chain like two bananas, as shown in Figure 3.

**Figure 4:**
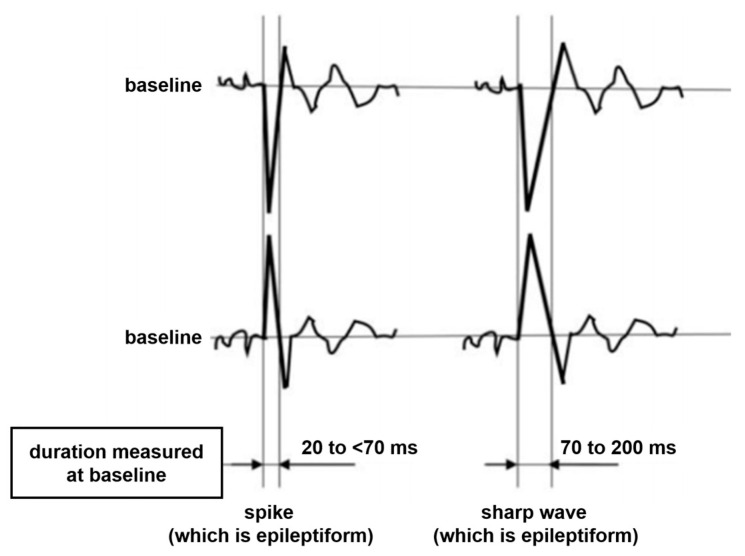
The patterns of the spike and the sharp wave in the bipolar montage.

In this study, the main detection targets are spikes and sharp waves, which are typical epilepsy abnormal brainwaves. In the bipolar montage, these two types of abnormal brainwaves have similar characteristics. Surges occur in adjacent channels. The peaks of the surges point tip to tip, and thus it can be identified at a glance. The only difference is that the two occur for different durations. Typically, spikes occur in 20 to 70 milliseconds and sharp waves occur in 70 to 200 milliseconds, as shown in Figure 4 [43].

### 3.3. Preprocessing

The sampling rate of all EDF(+) files provided by TCVGH medical doctors is not consistent. To make sure that every EDF(+) is at the same sampling rate, we check if the EDF(+) is at the most common sampling rate, which corresponds to 125 Hz in this study. If not, the sampling rate of EDF(+) data is downsampled to 125 Hz. The downsampling process consists of low-pass finite impulse response filtering followed by a sub-selecting mechanism. The difference between two adjacent timesteps is 8 milliseconds. Due to the short period of spikes and sharp waves, we divide the original EDF(+) data into 20 timesteps per sample which correspond to 160 milliseconds in a time window.

**Figure 5:**
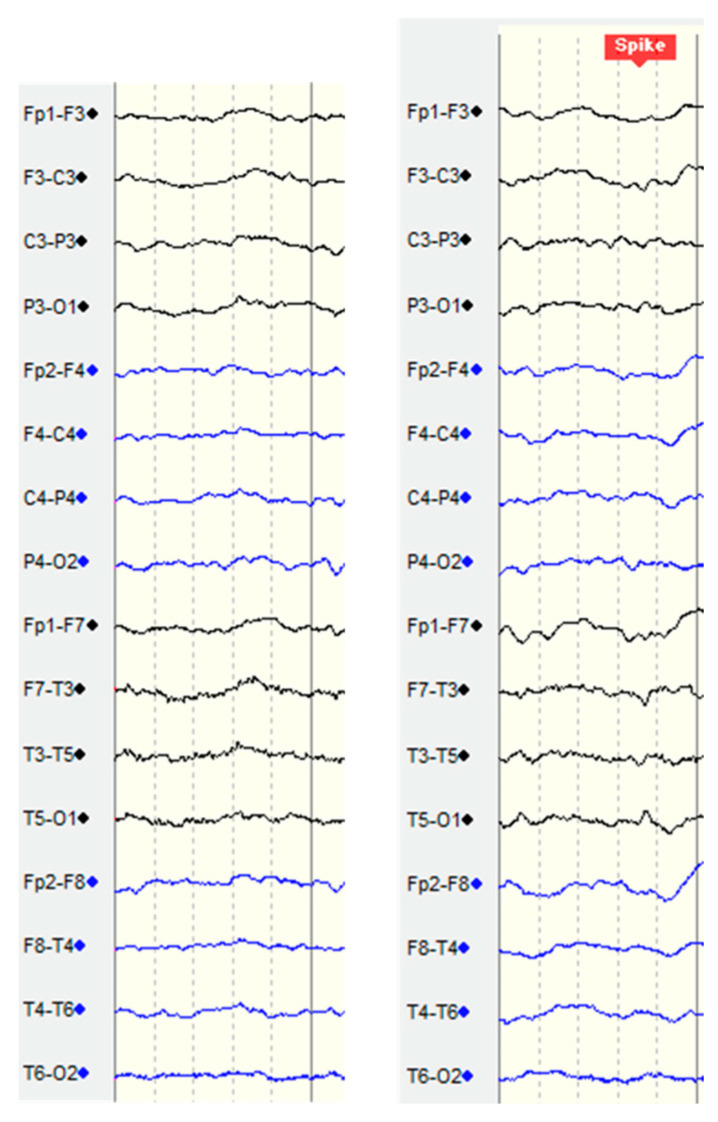
Example brainwaves with and without annotations provided by the TCVGH medical doctors. Black curves are the brainwaves collected from the left brain, whereas blue curves are those collected from the right brain.

When a sample contains the annotation provided by the TCVGH medical doctors, it is treated as an anomaly brainwave. Otherwise, it is marked as a normal brainwave. In the left figure of Figure 5, because the medical doctors did not annotate any labels in this window, we treat the samples gained from this period as normal brainwaves. In contrast, in the right figure of Figure 5, the medical doctors thought there was a spike in this period, so they marked the time point with the red box. Now, we get samples with two different classes. Next, we split all samples into three datasets: the training set, the validation set, and the testing set.

Some periods of EEG data may vibrate violently, and data from previous and subsequent time periods will not be on the same scale. We deal with this problem by normalizing every channel data individually to ensure that the values of each channel are on the same scale without losing the information about numerical level differences. We re-scale linearly the values of all channels to the range between zero and one by shifting the minimum and maximum values to zero and one, respectively. Consequently, the values of every sample data are on the same scale.

**Figure 6:**
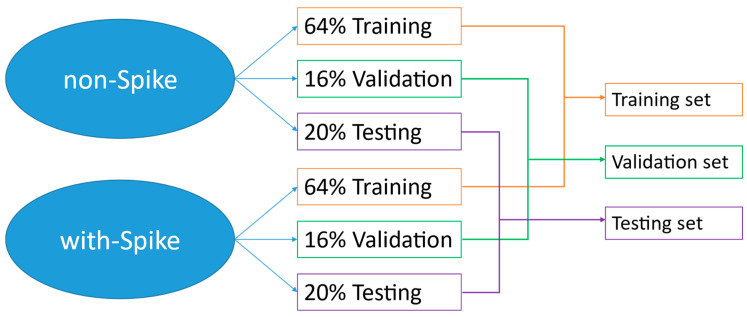
The process of dividing the data into training, validation, and testing sets to maintain the same proportion.

To maintain the same proportion as the original proportion of the number of different classes before dividing the data into training, validation, and testing sets, we divide each class into three sets in the same proportion individually and then merge them together, as shown in Figure 6. We first divide the original data into pseudo-training and testing sets according to the ratio of 8:2. Then, we further divide the pseudo-training set corresponding to 80% of the total data into the training and validation sets according to the ratio of 8:2. As a result, the training set accounts for 64%, the validation set accounts for 16%, and the testing set accounts for 20%.

### 3.4. Sampling Method

**Figure 7:**
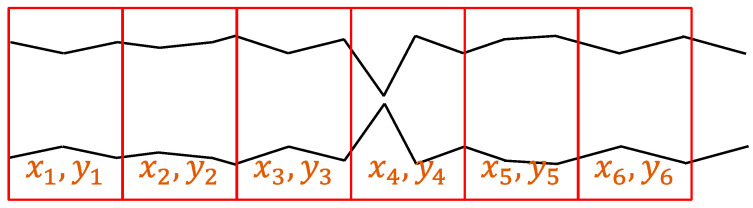
The cropping method. The EEG recording is partitioned consecutively into samples of 160 milliseconds without overlapping from the start to the end.

We crop the samples from the EEG recordings. There are 20 timesteps in one window, which corresponds to 160 milliseconds. If we discard some samples, the data cannot reflect the true situation of patients in ICUs. Thus, we crop and keep all the samples from 0 to the last second, as shown in Figure 7.

### 3.5. GRU-Based Model Architecture

**Figure 8:**
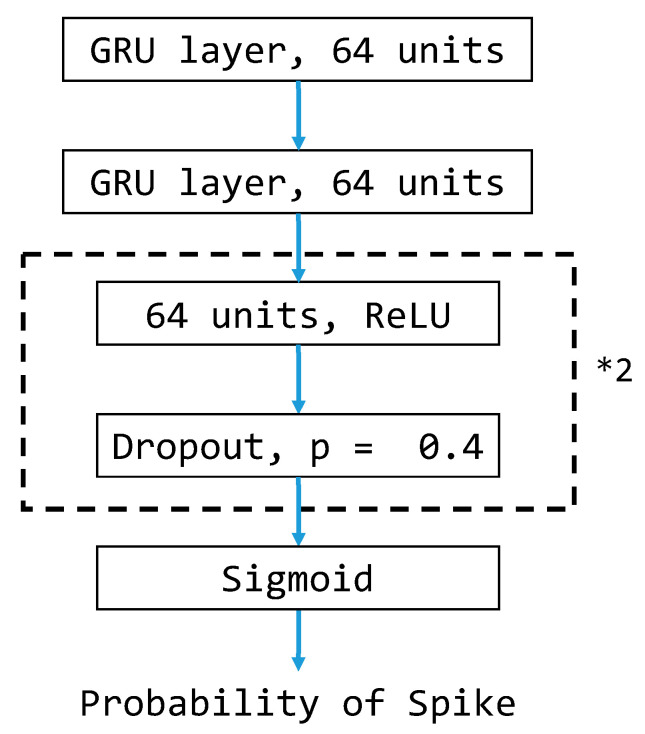
Proposed GRU-based model architecture. *2 means that the layers in the black dotted box are repeated twice in a cascade fashion in the model structure.

Because of the time dependency of EEG data, we adopt gated recurrent units (GRUs) in the proposed model to detect anomaly brainwaves in EEG recordings. GRU-based models are a kind of recurrent neural network and are particularly suited for performing predictions for time series data. The proposed model architecture is depicted in Figure 8 and will be the same for all experiments conducted in this study.

**Figure 9:**
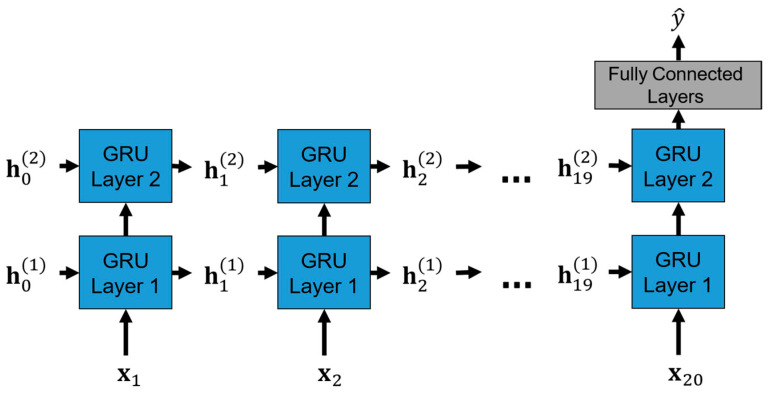
The unrolled operations of the proposed GRU-based model in the inference stage.

To be more specific, the operations of the proposed GRU-based model in the inference stage are unrolled and shown in Figure 9, where $x_{t}$ is the normalized 16-channel EEG recordings of the *t*-th timestep in one window. $h_{t-1}^{\left(k\right)}$ corresponds to the hidden states of the GRU layer *k* for the input $x_{t}$ that store the sequence information of the EEG recordings up to the $\left(t-1\right)$-th timestep. Each GRU layer consists of 64 GRU units involving the update and reset gating mechanisms that capture long- and short-term dependencies in EEG recordings. At the end of the 20th timestep, which corresponds to the last timestep in one window, the hidden states of the GRU layer 2, $h_{20}^{\left(2\right)}$, are used as the input of the fully connected layers with the ReLU activation functions to produce the final output prediction.

### 3.6. CNN-Based Model Architecture

**Figure 10:**
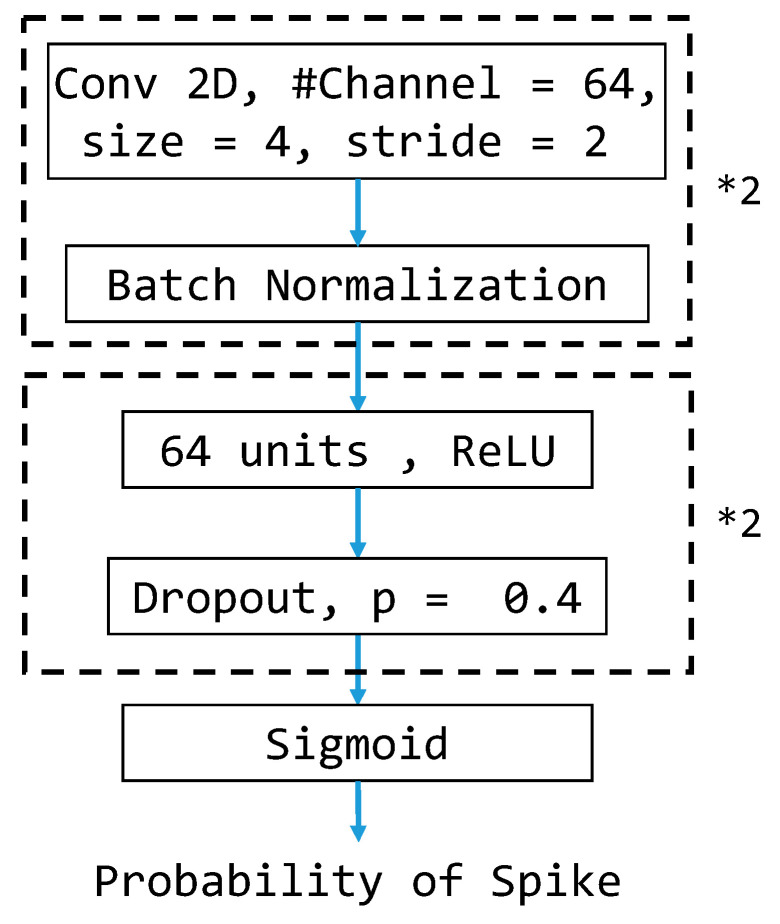
CNN-based model architecture for comparisons. *2 means that the layers in the corresponding black dotted box are repeated twice in a cascade fashion in the model structure.

Because of convolutional neural networks’ (CNNs’) powerful ability to extract features from every sample, we also test CNN-based models by treating the cropped brainwave samples as images. Thus, we construct the CNN-based model for the classification task. The architecture of the CNN-based models is depicted in Figure 10 and will be the same for all experiments conducted in this study. To be more specific, we treat each cropped brainwave sample as a 16 × 20 grayscale image fed into the CNN-based model. Each CNN layer consists of 64 convolutional filters that perform feature extraction to capture the local correlation within small patches and is followed by a batch normalization layer to provide suitable rescaling. The resulting features of the second CNN layer are used as the input of the fully connected layers with the ReLU activation functions to produce the final output prediction.

### 3.7. Class Weight

Adjusting the class weight in the training stage is a critical step in reducing the influence of the imbalance of the data. If the data are imbalanced, the models focus on the class with a larger amount. Models pay less attention to the class with a smaller amount. To reduce the influence, we adjust the class weight in the training stage. The class weight of each class is disproportionate to its amount so that models can pay attention to the pattern of both classes equally,

$${\mathrm{weight}}_{i}=\frac{\#\;\mathrm{of}\;\mathrm{total}\;\mathrm{training}\;\mathrm{data}}{\#\;\mathrm{of}\;\mathrm{training}\;\mathrm{data}\;\mathrm{from}\;\mathrm{class}\;i}\;,$$

where ${\mathrm{weight}}_{i}$ is the class weight assigned to class *i* during the model training.

### 3.8. Performance Metrics

In the stage of model training, we will use the validation set to choose the best model by monitoring the prediction performance after every training epoch. Since the detection of anomaly brainwaves is treated as a binary classification task, we may pick the epoch with the highest validation accuracy and retrieve the corresponding model as the final model. Due to the type of task, the accuracy of the model is the most important metric in our study. In the following section, we will use some metrics to quantify the performance of the deep learning models we build.

The confusion matrix, as shown in Table 2, can be used to provide the details of prediction results by the model. (1) defines the accuracy, which is the proportion of samples that are predicted correctly by the model. In medical applications, it shows the proportion of patients who are diagnosed with correct health status. (2) defines the sensitivity, which is the proportion of positive samples that are predicted as positive by the model and is an indicator to avoid a false negative. In medical applications, it shows the proportion of sick patients who are diagnosed with the disease. (3) defines the specificity, which is the proportion of negative samples that are predicted as negative by the model and is an indicator to avoid a false positive. In medical applications, it shows the proportion of people without the disease who are not diagnosed with it. (4) defines the balanced accuracy, which is the arithmetic mean of the sensitivity and specificity. Since the data are highly imbalanced between classes in this study, sensitivity, specificity, and balanced accuracy (BA) are more representative than accuracy for the performance evaluation of models.

(1) $$\mathrm{Accuracy}\;=\frac{\mathrm{TP}+\mathrm{TN}}{\mathrm{TP}+\mathrm{FN}+\mathrm{FP}+\mathrm{TN}}$$

(2) $$\mathrm{Sensitivity}\;=\frac{\mathrm{TP}}{\mathrm{TP}+\mathrm{FN}}$$

(3) $$\mathrm{Specificity}\;=\frac{\mathrm{TN}}{\mathrm{FP}+\mathrm{TN}}$$

(4) $$\mathrm{Balanced}\;\mathrm{Accuracy}\;\left(\mathrm{BA}\right)=\frac{\mathrm{Sensitivity}+\mathrm{Specificity}}{2}$$

## 4. Experiments and Results

This section illustrates experiments under different situations. We will use two different kinds of models with the same model complexity. After training, we pick the models corresponding to the highest validation accuracy or the highest validation balanced accuracy of the epoch and perform with the testing set to validate the model performance. We also attempt to achieve early detection in this study.

For all experiments, we use the same setting. Adam is adopted as the optimizer with a learning rate of 10^−4^. The batch size is 512, and the maximum number of epochs is 500. The cross-entropy loss with the adjusting class weights is used to guide the mode training. All experiments are conducted within the TensorFlow framework.

### 4.1. Experiment 1

**Figure 11:**
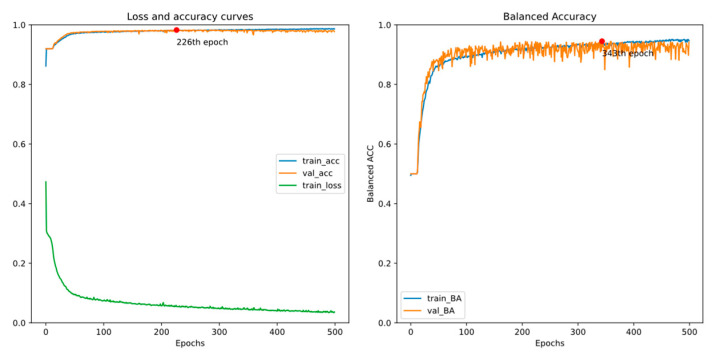
Training curves of the GRU-based model without adjusted class weights.

**Figure 12:**
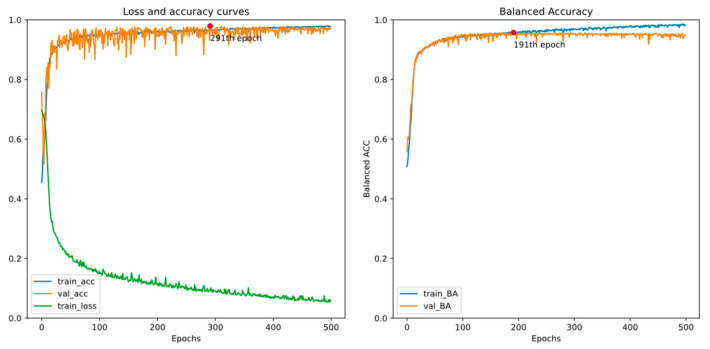
Training curves of the GRU-based model with adjusted class weights.

**Figure 13:**
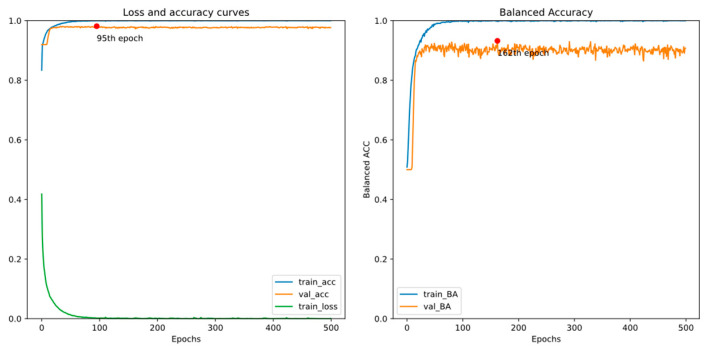
Training curves of the CNN-based model without adjusted class weights.

**Figure 14:**
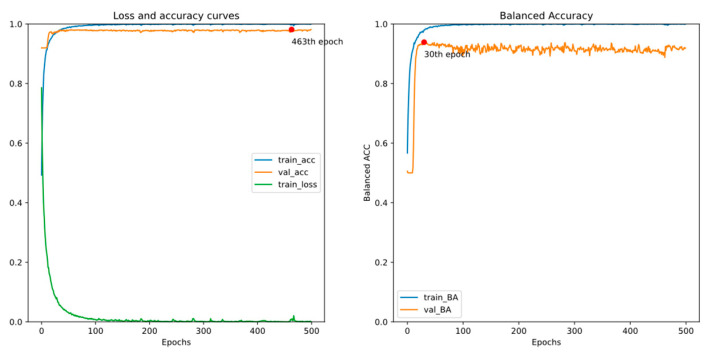
Training curve of the CNN-based model with adjusted class weights.

There are two EEG recordings containing a few spike annotations with a timeline error. Thus, we treat all samples from the two with a timeline error as negative in this experiment. Figure 11 and Figure 12 show the training curves of GRU-based models without and with adjusted class weights, respectively. Figure 13 and Figure 14 show the training curves of CNN-based models without and with adjusted class weights, respectively. All four figures show that the respective model learns well. The models are selected at different epochs indicated by the red points by monitoring the validation accuracy or the validation balanced accuracy.

Table 3 illustrates the performance of all models in this experiment. If we choose the final model by monitoring the validation accuracy, the sensitivity value is less than 90%. In addition, by monitoring validation balanced accuracy (BA), accuracy and specificity reduce a little, but sensitivity and BA, the metrics of interest in our study, may increase. Also, we can see that adjusting class weights can improve models’ performances and make them perform more stably in every metric.

### 4.2. Experiment 2

In Experiment 1, we treat all the samples cropped from the two recordings with timeline errors as negative. There are still some annotations that are not problematic in these two recordings, so we correct the samples corresponding to these annotations manually to the positive class. The sizes of training, validation, and testing set samples may change slightly. Eleven samples are fixed. The comparison of resulting model performances is shown in Table 4. We observe a similar result. The GRU-based model chosen by the validation BA with adjusted class weight performs the best in terms of the BA (94.66%) and sensitivity (93.12%) on the testing set.

### 4.3. Early Detection

**Figure 15:**
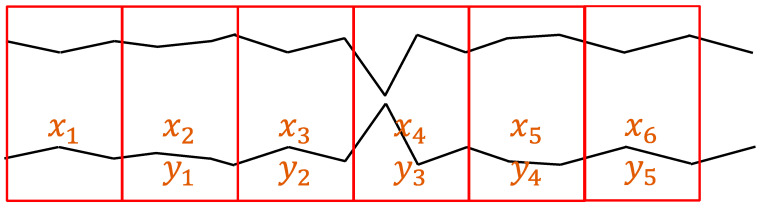
Cropping and labeling for one-window-early detection.

Following Experiment 2, in which the annotation errors are corrected, we attempt to perform early detection. To achieve early detection, we crop data and label them in different windows. Figure 15 shows the cropping and labeling example for one-window-early detection. We try early detection up to nine windows by monitoring validation BA to pick the final model. The setting we adopt for training the model uses the GRU-based model and adjusts the class weight during the training stage. Table 5 shows the testing result of early detection. The case with zero window early corresponds to the result of Experiment 2. We can see that the performance is maintained in one window early. As the number of windows increases, the model’s performance gradually differs from the original. Again, our primary concern is still balanced accuracy because of the imbalance of the data. We can find that as the number of windows increases, the value of this indicator has a gradual downward trend but is still in good shape (almost always above 90%).

## 5. Conclusions

We propose the GRU-based model with adjusted class weights to accomplish anomaly brainwave detection, which detects the existence of spikes and sharp waves in the EEGs of ICU patients. Unlike most other research, we adopt the bipolar montage in which medical doctors can easily find the anomaly events. The proposed GRU-based model can be used to monitor the brain activity of ICU patients more efficiently and cost-effectively. In clinical applications, the proposed GRU-based model can lighten the workload of medical staff in ICUs. Despite the data imbalance, our models’ sensitivity, specificity, and balanced accuracy are still all above 90%. These three metrics can represent the actual model performance. Although there is room for better sensitivity, our models can ease the burden on the medical staff in ICUs.

In addition to in-time detection, we also attempted early detection. In units of one window, we tried from one to nine windows and observed what pattern the models learned. As the number of windows increases, the balanced accuracy has a gradual downward trend but is still almost always above 90%. This justifies that the proposed GRU-based model with adjusted class weights has great potential in clinical applications.

In this study, we also include the CNN-based model architecture for comparisons. The methods of CNN-based models and GRU-based models can both be used for offline detection. Hence, we conduct the performance comparison for these two methods for offline detection. It turns out that the performance of the GRU-based model is better than that of the CNN-based model for offline detection in this empirical study. Furthermore, the GRU-based model can be used for real-time detection, but the CNN-based model cannot be used for such an application. Hence, the method of the GRU-based model is the suitable approach for offline and real-time detection.

The current models for automated anomaly detection are developed for patients’ data collected only in a medical center, i.e., TCVGH. Their detection performance in clinical applications may be degraded when they are applied to different medical centers and/or regional hospitals. To ensure satisfying detection performance across medical institutions, we may need to collaborate with them and collect their ICU patients’ data to fine-tune or re-train the detection models. Another way is to develop federated learning-based models so that the detection models can be jointly trained in a decentralized way while preventing the violation of patient privacy [44].

## Figures and Tables

**Table 1:** Comparison of existing works.

Objective	Method	ICU Patients	Montage	Window Size	Performance
transient event classification [18]	mimetic analysis, power spectral analysis	No	bipolar	355 ms	87.38% accuracy
spike detection [19]	template matching	No	average reference, bipolar	5.12 s	92.6% selectivity
IED detection [21]	wavelet analysis	No	average reference	3 s	90.5% accuracy
epileptic activity classification [23]	artificial neural network	No	bipolar	355 ms	84.48% accuracy
spike detection [28]	deep learning	No	average reference	0.5 s	0.947 AUC
spike detection (ours)	deep learning	Yes	bipolar	160 ms	94.66% balanced accuracy

**Table 2:** Confusion matrix for performance evaluation of models.

		Predicted Class	
		Positive	Negative
**True** **Class**	**Positive**	True Positive (TP)	False Negative (FN)
	**Negative**	False Positive (FP)	True Negative (TN)

**Table 3:** Comparison of the testing performance (%) in Experiment 1.

	Model	By	Accuracy	Sensitivity	Specificity	BA
**No Class Weight**	**GRU**	**Acc**	**98.19**	85.24	**99.33**	92.28
		**BA**	97.32	91.54	97.83	**94.68**
	**CNN**	**Acc**	97.93	81.30	99.38	90.34
		**BA**	97.21	87.80	98.04	92.92
**W** **ith Class Weight**	**GRU**	**Acc**	97.83	87.99	98.69	93.34
		**BA**	95.95	**93.11**	96.19	**94.65**
	**CNN**	**Acc**	97.61	81.10	99.05	90.08
		**BA**	97.15	90.16	97.76	93.96
**Z** **ero-rule Baseline**			91.95	0.00	100.00	50.00

**Table 4:** Comparison of the testing performance (%) in Experiment 2.

	Model	By	Accuracy	Sensitivity	Specificity	BA
**No Class Weight**	**GRU**	**Acc**	**98.02**	82.12	**99.41**	90.77
		**BA**	97.58	90.18	98.23	94.20
	**CNN**	**Acc**	97.80	85.27	98.90	92.08
		**BA**	97.13	88.21	97.92	93.06
**W** **ith Class Weight**	**GRU**	**Acc**	97.93	85.27	99.04	92.15
		**BA**	95.95	**93.12**	96.19	**94.66**
	**CNN**	**Acc**	97.94	83.50	99.21	91.35
		**BA**	97.61	89.98	98.28	94.13
**Z** **ero-rule Baseline**			91.94	0.00	100.00	50.00

**Table 5:** Comparison of the testing performance for early detection.

# of Windows Early										
	0	1	2	3	4	5	6	7	8	9
**Accuracy**	95.95	93.38	93.65	94.26	93.15	94.18	94.96	93.65	94.97	96.57
**Sensitivity**	93.12	93.59	90.82	93.22	92.37	91.56	89.36	92.33	85.54	82.04
**Specificity**	96.19	93.36	93.83	94.34	93.21	94.35	95.31	93.74	95.61	97.49
**Balanced Accuracy**	94.66	93.48	92.33	93.78	92.79	92.95	92.34	93.03	90.57	89.76
**Zero-rule Accuracy**	91.94	93.82	93.79	93.69	93.77	93.99	94.04	93.80	93.64	94.08
**Zero-rule Baseline**	Sensitivity: 0.00			Specificity: 100.00			Balanced Accuracy: 50.00			

## Data Availability

The data in the present study are available upon request from the corresponding author.
